# Association of *FTO* Polymorphisms with Early Age of Obesity in Obese Italian Subjects

**DOI:** 10.1155/2012/872176

**Published:** 2012-02-20

**Authors:** Federica Sentinelli, Michela Incani, Federica Coccia, Danila Capoccia, Valentina Maria Cambuli, Stefano Romeo, Efisio Cossu, Maria Gisella Cavallo, Frida Leonetti, Marco Giorgio Baroni

**Affiliations:** ^1^Endocrinology and Diabetes, Department of Medical Sciences, University of Cagliari, Policlinico Universitario, 09042 Cagliari, Italy; ^2^Department of Clinical Sciences, University of Rome “Sapienza”, 00161 Rome, Italy; ^3^Department of Molecular and Clinical Medicine, Sahlgrenska Center for Cardiovascular and Metabolic Research, University of Gothenburg, 431 45 Gothenburg, Sweden; ^4^Department of Medical and Surgical Sciences, University of Magna Graecia, 88100 Catanzaro, Italy; ^5^Department of Clinical and Medical Therapy, University of Rome “Sapienza”, 00185 Rome, Italy

## Abstract

Obesity is recognized as a major health problem worldwide. Genetic factors play a major role in obesity, and genomewide association studies have provided evidence that several common variants within the fat mass- and obesity-associated (*FTO*) gene are significantly associated with obesity. Very limited data is available on *FTO* in the Italian population. Aims of our study are to investigate: (1) the association of *FTO* gene SNPs rs9939609 and rs9930506 with body mass index (BMI) and obesity-related parameters in a large cohort (*n* = 752) of Italian obese subjects; (2) the association between the two *FTO* SNPs and age of onset of obesity. Our results demonstrate a strong association between *FTO* SNPs rs9939609 (*P* < 0.043) and rs9930506 (*P* < 0.029) with BMI in the Italian population. *FTO* rs9930506 was significantly associated with higher BMI in a G allele dose-dependent manner (BMI + 1.4 kg/m^2^ per G allele). We also observed that the association with BMI of the two *FTO* variants varied with age, with the carriers of the risk alleles developing an increase in body weight earlier in life. In conclusion, our study further demonstrates a role of the genetic variability in *FTO* on BMI in a large Italian population.

## 1. Introduction

Obesity is considered a worldwide epidemic in modern societies, affecting all ages, genders, and ethnic groups. Increased adiposity is due to the interaction between a genetic background and environmental factors, particularly excessive food intake and reduced physical activity. Although the obesity epidemic has been certainly driven by lifestyle and environmental changes, it is also clear that there is individual variation in response to these factors, suggesting a strong genetic predisposition. In recent years, of the many genes studied for obesity, the *FTO* (fat mass- and obesity-associated) gene has been proven consistently to be associated with increased body mass index (BMI). Since its discovery in 2007 in genomewide association studies for type 2 diabetes [1], several other studies have confirmed the association in different populations [2–4]. Of the many found associated, the *FTO* rs9939609 single nucleotide polymorphism (SNP), located in the first intron, is of particular interest since it was the first to be found to be associated with obesity and has been constantly replicated through independent studies of large Caucasian populations [2, 3]. It was estimated that each copy of the highly frequent (40–50% in the general population) *FTO* rs9939609 minor allele (i.e., A allele) corresponds to approximately 1.5-kg heavier body weight [1].

*FTO* is mainly expressed in the hypothalamus, and it may play important roles in the management of energy homeostasis [5], nucleic acid demethylation, and in the regulation of body fat masses by lipolysis [6].

Other than BMI, *FTO* gene SNPs have been shown to associate with a number of metabolic-related traits, such as higher fasting insulin, glucose, triglycerides and lower HDL cholesterol [7], waist circumference [8, 9], and body weight [10].

So far, very limited data is available on the relationship between *FTO* gene and obesity in the Italian adult population. The only study published in a cohort from Sardinia [10] showed association between the *FTO *rs9939609 SNP and BMI, total body weight, and hip circumference. Furthermore, a second *FTO *SNP, the rs9930506, showed in this population an even stronger association [10] compared to the rs9939609 SNP. It should be pointed out that this population is from Sardinia, an Italian island that shows peculiar genetic characteristics that may differ from the rest of the Italian population [11]. 

Various studies have investigated the effect of *FTO* variants on BMI and weight in a longitudinal perspective, and whether it influences weight gain during adult life [12, 13]. Interestingly, a stronger correlation between BMI and *FTO* single nucleotide polymorphisms is most commonly seen in cohorts of children and young adults [14, 15].

Aims of our study are therefore: (1) to investigate the association of the *FTO* gene SNPs rs9939609 and rs9930506 with BMI and obesity-related parameters in a large cohort (*n* = 752) of Italian obese population; (2) to examine the association between the two *FTO* SNPs and age of onset of obesity.

## 2. Methods

### 2.1. Study Group

A total of 950 subjects were studied. 752 consecutive unrelated obese (BMI ≥ 30 kg/m^2^) Caucasians were recruited from the Day-Hospital of the Department of Clinical Sciences, University of Rome “Sapienza”. They underwent complete medical evaluation and a standard 75 g oral glucose tolerance test (OGTT) with measurements of glucose and insulin at baseline and after 30, 60, 90, and 120 minutes. All individuals provided informed consent prior to inclusion in the study. The study was approved by the local research ethics committee.

Nonobese subjects (*n* = 198) were recruited from subjects participating in a community-based health screening. All subjects were unrelated, and the only inclusion criteria in the control group for obesity was a BMI < 27 kg/m^2^. In nonobese subjects a complete medical history was obtained together with laboratory parameters including total cholesterol, HDL, LDL, triglycerides, blood glucose, and fasting plasma insulin.

In all subjects diagnosis of type 2 diabetes was based on either fasting plasma glucose ≥ 7.0 mmol/L (126 mg/dL), or plasma glucose ≥ 11.1 mmol/L (200 mg/dL) 2 hours after a 75 g glucose load during the OGTT. Also a previous diagnosis of diabetes and/or a history of hypoglycaemic treatment were considered.

### 2.2. Biochemistry

Glucose, insulin, cholesterol, HDL-cholesterol, and triglycerides (TGs) were measured as previously described [16], after an overnight fast. Glucose levels below 100 mg/dL, HDL > 40 mg/dL for men and >50 mg/dL for women, and triglycerides < 150 mg/dL were taken as normal limits.

Serum alanine (ALT) and aspartate aminotransferase (AST) levels in fasting subjects were assayed using a Hitachi 737 analyzer (Boehringer-Mannheim Diagnostics, Indianapolis, IN). Homeostasis model assessment of insulin resistance (HOMA-IR) and insulin sensitivity index (ISI) indices were calculated as previously shown by Matthews et al. [17] and Matsuda and Defronzo [18]. 

### 2.3. Genotyping Assay

Genomic DNA samples were extracted from peripheral blood, using a modified salting out procedure from Miller et al. [19].

In this study, we have used locked nucleic acid (LNA) hybridization probes (fluorometric method) as allele-specific tools in genotyping assays in order to analyze each SNPs (rs9930506 and rs9939609). Real time PCR was performed using iQ5 Multicolor Real-Time (Bio-Rad Laboratories), and data analysis was conducted with the corresponding software interface. In a 25 *μ*L reaction volume 300 nM of each PCR primer, 280 nM of each LNA probe, 1X iQ Supermix (Bio-Rad Laboratories), and 10 ng of genomic DNA were added.

PCR primers and LNA probes were designed and synthesized by Sigma-Aldrich Life Science (further data on primers and probes are available upon request).

Some genomic DNA samples used for real-time PCR optimization were sequenced to validate the observed allelic discrimination curves and genotypes.

### 2.4. Statistical Analysis

Categorical variable distributions were compared by the Pearson *χ* ^2^. Differences between continuous variables across the genotype classes were evaluated by ANOVA including gender, age, and BMI as covariates. Skewed variables were logarithmically transformed prior to entering the analyses. Linkage disequilibrium between the rs9939609 and rs9930506 SNPs was assessed by calculating the disequilibrium statistics Δ [20] and D′ [21]. The sign of D′ (positive or negative) depends on the arbitrary choice of the alleles paired at the two loci and indicates whether the same or opposite allelic association is present. The differences in the variables within the same group before and after bariatric surgery were compared with paired-samples statistics. All statistical analyses were performed with SPSS 17.0 statistical package.

## 3. Results

### 3.1. Characteristics of the Study Population

Clinical characteristics of the study subjects stratified by classes of BMI (group A, nonobese: BMI < 27 kg/m^2^; group B, class I/II obese: BMI = 30–39.9 kg/m^2^; group C, class III obese: BMI ≥ 40 kg/m2) are reported in Table 1. Overall, 752 (79%) of the 950 study subjects were obese (422 with class III obesity), whereas 209 (22%) subjects had T2D. 

Generally, as expected, many clinical and biochemical data worsened significantly with increasing BMI, with class III obese subjects showing the highest levels of fasting blood glucose and insulin, lipids, transaminases, HOMA-IR, and lower values of ISI. In the control normal-weight group all parameters were within normal range, with only 5 subjects with diabetes.

### 3.2. *FTO* Allele Frequencies and Linkage Disequilibrium

In the whole population risk-allele frequencies of the rs9939609 (A) and rs9930506 (G) *FTO* gene were 0.48 and 0.50, respectively, similar to HapMap (haplotype map of the human genome) population frequencies for rs9939609 (A) allele in CEPH Europeans and for rs9930506 (G) allele in CEU Europeans.

The two SNPs showed a strong linkage disequilibrium (Δ = 0.88, D′= 0.90, *P* < 0.0001). Nevertheless, because of a different effect observed in Italian subjects [10], we analyzed the two variants separately and as haplotypes in our study cohort.

### 3.3. Association Study of the rs9939609 and rs9930506 Polymorphisms with BMI and T2D

Table 2 shows the association of the 2 *FTO* variants with obesity; the rs9939609 A-allele frequency and rs9930506 G-allele frequency in the obese subjects were significantly higher than in the lean group (*P* < 0.027 and *P* < 0.013, resp.). When the association was adjusted by age and sex, significance was maintained, although at lower *P* values, suggesting a strong effect of these parameters (*P* < 0.038 and *P* < 0.015, resp.). 

When we stratified for BMI levels, no significant difference in rs9939609 A-allele frequency was observed between class I/II obesity and class III obesity (Table  2(a)). Similar results were observed for rs9930506 G-allele frequency between class I/II obesity and class III obesity (0.51 and 0.52, resp.) (Table  2(b)). With reference to the rs9939609 (A) allele we should point out that we did not observe a significant difference between controls and class I/II obesity. This may be explained by the fact that this SNP has a weaker effect in Italian population, as also demonstrated by the study of Scuteri et al. [10]. Conversely, rs9930506 was, in the same population, significantly associated with class I/II obesity.

We next tested the association with T2D. A-allele frequencies for rs9939609 polymorphism were not significantly different between T2D and nondiabetic individuals (0.51 and 0.50, resp. *P* = NS); similarly, no significant differences between T2D and nondiabetic subjects were observed for the rs9930506 G-allele (0.51 and 0.52, resp. *P* = NS).

We then compared other clinical parameters according to genotype classes for both SNPs. There were no significant differences between the three genotypes in fasting blood glucose and insulin levels, plasma concentration of ALT and AST levels, total cholesterol, HDL- and low density lipoprotein- (LDL-)cholesterol, and circulating triglycerides. We also tested for an association with surrogate indices of insulin resistance (homeostatic model assessment of insulin resistance and Matsuda insulin sensitivity index), but no differences were found for both SNPs (data not shown). The only associations were observed between the *FTO *gene SNPs and BMI, sex, and age (Table 3). Interestingly, in our Italian population the rs9930506 showed the strongest association with BMI (*P* < 0.029) and waist circumference (*P* < 0.006). Specifically, homozygotes for the G-allele of this SNP were 2.8 BMI units heavier than homozygotes for the A-allele (Table 3). Moreover, the rs9930506 G allele was significantly associated with higher BMI, in a G allele dose-dependent manner (BMI + 1.4 kg/m^2^ per G allele). A similar trend was observed for homozygotes for the A-allele of rs9939609 polymorphism although with a weaker significance (*P* < 0.043). 

Homozygotes for the G-allele of the rs9930506 SNP were also significantly younger than homozygotes for the A-allele (*P* < 0.013) (Table 3), and a similar trend was observed for homozygotes for the A-allele of rs9939609 polymorphism (*P* < 0.006) (Table 3). 

### 3.4. Association Study of the rs9939609 and rs9930506 Polymorphisms with Age

We further evaluated the distribution of the rs9939609 and rs9930506 *FTO* SNPs genotypes according to age, stratifying the study population in younger subjects (<46 years of age, median of the whole population) and older subjects (≥46 years of age). The rs9930506 G-allele frequency in younger subjects was significantly higher than in older group (0.54 versus 0.46, *P* < 0.004) and similarly for rs9939609 A-allele frequency (0.53 versus 0.45, *P* < 0.002). Further stratification in quartiles of age showed that subjects in the 75th quartile (>55 years) had a much lower frequency of “at risk” *FTO *alleles (rs9939609 (A) = 0.42 and rs9930506 (G) = 0.44) compared to subjects in the 25th quartile (age > 35 years, 0.53 A allele and 0.54 G allele), a difference that was highly significant (*P* < 0.01). Since class III obese were younger they might explain most of the difference of frequency distribution of the rs9930506 G-allele and of the rs9939609 A-allele between younger and older subjects. In order to analyze this point we have performed two separate analyses in our population: (1) we repeated the same analyses in Table 3 excluding the class III obese subjects, and association with age was still highly significant (data not shown, *P* < 0.01); (2) we also repeated the same analyses in the three subgroups, selecting age-matched subjects from the three BMI classes (Group A mean age  49 ± 10  years, Group B mean age  48 ± 14  years, Group C mean age  49 ± 10  years, *P* = NS).

As shown in Table 4 the association between *FTO* SNPs and age remained highly significant (*P* < 0.01).

### 3.5. Haplotypes Analyses from rs9939609 and rs9930506 Polymorphisms

Analyses in the haplotypes groups derived from rs9939609 and rs9930506* FTO* genetic polymorphisms confirmed the lack of association of the *FTO *variants with fasting blood glucose and insulin levels, plasma ALT and AST levels, total cholesterol, HDL- and low density lipoprotein- (LDL-) cholesterol, triglycerides, HOMA-IR, and ISI indices (data not shown). However the AG haplotype (homozygous carriers of “at risk” alleles) was associated with increased BMI (*P* < 0.026) and waist circumference (*P* < 0.003) which is consistent with the findings observed for the two SNPs separately (data not shown). Furthermore, AG haplotype carriers were significantly younger than homozygotes for the TA haplotype (*P* < 0.014) (data not shown) confirming the results of the two SNPs analyzed individually.

### 3.6. Multivariate Analyses of *FTO* Polymorphisms

Due to the strong linkage disequilibrium between rs9939609 and rs9930506 *FTO *genetic polymorphisms, only rs9930506 (the most significant) SNP was considered for further analyses. Since univariate analyses showed a strong association of the rs9930506 *FTO *only with BMI, age, and waist, we entered in a multiple regression model these three variables. Multiple regression analysis with BMI as a dependent variable revealed that the rs9930506 *FTO *genetic polymorphisms are associated with body mass index (*P* < 0.029), independently of sex (*P* < 0.036) and age (*P* < 0.001).

## 4. Discussion

### 4.1. Association of *FTO* rs9939609 and rs9930506 Polymorphisms with BMI

Our results demonstrate in the Italian population a strong association between rs9939609 and rs9930506 SNPs of the *FTO *gene and BMI and waist circumference, in concordance with previously published studies in other European populations [1–3, 10]. Of the 2 SNPs, rs9930506 was the most strongly associated with BMI in our Italian population, confirming the only previous observation [10]. Furthermore, the G allele of rs9930506 was significantly associated with higher BMI in a G allele dose-dependent manner (BMI + 1.4 kg/m^2^ per G allele).

### 4.2. Association of *FTO* rs9939609 and rs9930506 Polymorphisms with Age

We also observed that the associations of *FTO* rs9939609 and rs9930506 variants on body size varied with age. Particularly, the at-risk allele frequencies for both SNPs were significantly higher in younger than in older subjects, suggesting that carriers of the risk allele develop an increase in body weight earlier in life.

There have been only a few other studies exploring the association between *FTO *gene and BMI during the life course [12, 13]. In the study by Hardy and coworkers a longitudinal pattern of association between rs9939609 *FTO *genetic variant and BMI across childhood, adolescence, and adulthood was observed [13]. In particular, the associations with this *FTO *variant strengthened during childhood and adolescence, peaked in early adulthood, and then weakened in adult age (after 43 years). It can be hypothesized that the effect of *FTO *on body composition may be less severe in adult life when weight gain may be more strongly determined or modified by psychosocial or environmental influences [22] compared to younger ages.

### 4.3. Lack of Association of *FTO* Polymorphisms with Metabolic Parameters and T2D

At variance with previous studies [7–9], in our population *FTO *gene polymorphisms were associated only with an increased BMI, but not with metabolic parameters such as lipids, impaired glucose tolerance, or insulin resistance. One possible explanation for these discrepancies may be ascribed to the relatively young age of our population (median 46 years), with >50% of carriers of the *FTO *risk allele that are under the median age and with a significant proportion of carriers that are below the 25th percentile of age (<35 years) of our cohort. Thus, these subjects may have not yet developed age-related metabolic abnormalities.

We also tested a possible association with T2D, but no significant differences between T2D and nondiabetic subjects were observed for the *FTO *SNPs. It has been previously observed that the association of the SNPs (located in the first intron) of the *FTO *gene with T2D was abolished after adjustment for BMI, indicating that the impact of *FTO *on T2D was primarily due to its association with BMI [1]. Our study population presented a prevalence of obesity of ~79%, and this might explain the absence of association of *FTO *SNPs with T2D.

It is unclear how *FTO *increases the susceptibility to develop overweight and obesity. There are no obvious evidences suggesting that rs9939609 or rs9930506 are the causal variants, and, indeed, there are many other variants elsewhere in the gene or control elements of other genes that are in complete linkage disequilibrium with the *FTO* locus. However, based on the rapid fall-off of linkage disequilibrium with other SNPs beyond 47 kb, it has been concluded that the functional variant is likely to lie within this area of the *FTO *gene [1].

Through the use of bioinformatics and recombinant functional studies it has been recently demonstrated that the *FTO *gene encodes a nucleic acid demethylase [23]. Demethylation of DNA is essential for repairing exocyclic DNA lesions, which, if left unchecked, can lead to permanent, and sometimes deleterious, sequence changes. It is also conceivable that the nucleic acid demethylation activity of *FTO *on DNA might regulate the expression of genes involved in metabolism and that dysregulation of this process might lead to obesity at an epigenetic level [24].

## 5. Conclusion

*FTO* is the first and the most robust obesity susceptibility gene of the GWAS era. Although its effect size is modest, one should not underestimate the relevance of *FTO* at the population level. The observed association is common and consistent across multiple ethnic groups and could be influencing BMI of up to half the world's population.

Our data confirm all these findings. In particular, we observe that the association between *FTO *gene and BMI strongly influences the age of onset of obesity, with the carriers of the “at risk” alleles showing a significantly higher prevalence in younger age. In conclusion, our study shows a role of the genetic variability in *FTO *in the modulation of BMI in a large Italian population.

## Figures and Tables

**Table 1:** Clinical characteristics of study subjects stratified for BMI.

*N*	Group A Nonobese 198	Group B Class I/II obese 330	*P * A versus B	Group C Class III obese 422	*P * A versus C	*P * B versus C
Women/men	159/39	222/108	<0.001	303/119	<0.01	NS
Age (yr)	49 (45–55)	48 (34–58)	NS	43 (35–51)	<0.001	0.005
Body mass index (kg/m^2^)^a^	23.0 (21.3–24.7)	35.7 (32.9–38.1)	<0.001	46.2 (42.8–51.1)	<0.001	<0.001
WAIST (cm)	95.5 (91–100)	112 (105–120)	0.045	132.5 (123–142)	<0.001	<0.001
SBP (mm/Hg)	120 (115–135)	130 (120–140)	NS	130 (120–140)	NS	NS
DBP (mm/Hg)	80 (70–86)	80 (75–90)	NS	85 (80–90)	<0.001	<0.001
Triglycerides (mg/dL)	85.5 (65.6–120.3)	127.3 (85.9–181)	<0.001	125 (88.6–163)	<0.001	NS
Total cholesterol (mg/dL)	209 (187.2–234)	203 (178–234)	NS	195 (170–218.3)	<0.001	<0.003
HDL cholesterol (mg/dL)	59 (50–67)	47.3 (40.3–56.6)	<0.001	45.8 (39.8–53)	<0.001	<0.033
LDL cholesterol (mg/dL)	126 (108–147)	124.2 (105–149)	NS	115 (95.1–139.6)	<0.003	<0.021
ALT (U/L)	23 (13.1–34.5)	27.5 (19.2–40)	NS	29.1 (20.7–45.6)	NS	NS
AST (U/L)	20 (18–25)	20 (16–27)	NS	20 (16–28)	NS	NS
Fasting blood glucose (mg/dL)	92 (84–100)	93 (82–118)	NS	95 (85–111)	<0.001	NS
Fasting blood insulin (*μ*U/mL)	9.9 (7–15.2)	18.8 (13–30)	<0.001	29 (19.5–47.3)	<0.001	<0.001
HOMA-IR (U)	2 (1.5–3.3)	3.9 (2.6–6.7)	<0.001	6.2 (4.1–10.7)	<0.001	<0.001
ISI (U)	4.6 (2.9–7.8)	2.8 (1.8–4.4)	<0.001	1.9 (1.2–2.8)	<0.001	<0.001
Type 2 diabetes *n* (%)	5 (2.5)	101 (30.6)	<0.001	103 (24.4)	<0.001	<0.046

^a^BMI was calculated as body weight (kg)/height (m^2^). Values are medians and interquartile ranges. Abbreviations: SBP, systolic blood pressure; DBP, diastolic blood pressure; HDL, high density lipoprotein; LDL, low density lipoprotein; ALT, alanine transaminase; AST, aspartate transaminase; HOMA-IR, homeostatic model assessment of insulin resistance; ISI, insulin sensitivity index. Non-obese: BMI < 27 kg/m^2^; class I/II obese: BMI = 30-39.9 kg/m^2^; class III obese: BMI ≥ 40 kg/m^2^.

**Table:** (a)

		Genotype *n* (%)			A-allele frequency (%)	*P*	*P*_adj_
	*n*	TT	TA	AA			
*Stratified on BMI*							
Nonobese	198	68 (34.3)	90 (45.5)	40 (20.2)	43.0	<0.027	<0.038
Obese	752	195 (25.9)	357 (47.5)	200 (26.6)	50.3		
*Stratified on BMI*							
Nonobese	198	68 (34.3)	90 (45.5)	40 (20.2)	43.0		
Class I/II obese	330	88 (26.7)	152 (46.1)	90 (27.2)	50.3	<0.066*	<0.075*
Class III obese	422	107 (25.4)	205 (48.6)	110 (26.0)	50.4	<0.038*	<0.057*

**Table:** (b)

		Genotype *n *(%)			G-allele frequency (%)	*P*	*P*_adj_
	*n*	AA	AG	GG			
*Stratified on BMI*							
Nonobese	198	67 (33.8)	90 (45.5)	41 (20.7)	43.4		
Obese	752	190 (25.3)	346 (46.0)	216 (28.7)	51.7	<0.013	<0.015
*Stratified on BMI*							
Nonobese	198	67 (33.8)	90 (45.5)	41 (20.7)	43.4		
Class I/II obese	330	86 (26.1)	149 (45.2)	95 (28.7)	51.4	<0.043*	<0.038*
Class III obese	422	104 (24.6)	197 (46.7)	121 (28.7)	52.0	<0.018*	<0.019*

Data are * n* (%). *P*_adj_ values were calculated using logistic regression analysis adjusted for age and sex. Nonobese: BMI < 27 kg/m^2^; class I/II obese: BMI = 30–39.9 kg/m^2^; class III obese: BMI ≥ 40 kg/m^2^. *versus nonobese

**Table 3:** Association of rs9930506 and rs9939609 SNPs with age, BMI, and waist.

SNP	Genotype Class (*n*.)	Age (years)	BMI (kg/m^2^)	Waist (cm)
rs9930506	AA (257)	47 ± 14	37.2 ± 11.4	117.8 ± 18.7
	GA (436)	45 ± 13	38.6 ± 10.4	124.2 ± 18.5
	GG (257)	44 ± 13	40 ± 9.6	126.7 ± 15.2
	*P*	<0.013*	<0.029**	<0.006*

rs9939609	TT (263)	47 ± 14	37.3 ± 11.4	118.4 ± 19.5
	TA (448)	45 ± 13	38.6 ± 10.4	125 ± 18.2
	AA (239)	43 ± 13	39.4 ± 9.8	125 ± 14.5
	*P*	<0.006*	<0.043**	<0.025*

All values are means ± standard deviations. BMI: body mass index,* n.*: number of subjects. *P values were calculated using a linear regression model including gender, age, and BMI as covariates. **Analysis adjusted for gender and age.

**Table 4:** Association of rs9930506 and rs9939609 SNPs with age, BMI, and waist in age-matched subjects selected from the three BMI classes.

SNP	Genotype Class (n.)	Age (years)	BMI (kg/m^2^)	Waist (cm)
rs9930506	AA (224)	50 ± 11	36.1 ± 11.2	116.1 ± 18.8
	GA (364)	48 ± 10	37.6 ± 10.6	124.8 ± 18.6
	GG (215)	46 ± 12	38.3 ± 9.5	125.5 ± 15.1
	*P*	<0.010*	<0.022**	<0.005*

rs9939609	TT (224)	50 ± 11	36.1 ± 11.3	116.8 ± 20
	TA (364)	48 ± 11	37.5 ± 10.5	125.1 ± 18.2
	AA (215)	46 ± 12	38.2 ± 9.6	124 ± 14.6
	*P*	<0.011*	<0.038**	<0.012*

All values are means ± standard deviations. BMI: body mass index; *n.*: number of subjects. *P values were calculated using a linear regression model including gender, age, and BMI as covariates. **Analysis adjusted for gender and age.
